# Hypercholesterolemia Tunes Hematopoietic Stem/Progenitor Cells for Inflammation and Atherosclerosis

**DOI:** 10.3390/ijms17071162

**Published:** 2016-07-19

**Authors:** Xiaojuan Ma, Yingmei Feng

**Affiliations:** 1 Beijing Key Laboratory of Diabetes Prevention and Research, Lu He Hospital, Capital Medical University, Beijing 101149, China; maxiaojuans@163.com; 2 Department of Endocrinology, Lu He Hospital, Capital Medical University, Beijing 101149, China

**Keywords:** hematopoietic stem/progenitor cells, hypercholesterolemia, reactive oxygen species, cholesterol efflux, atherosclerosis

## Abstract

As the pathological basis of cardiovascular disease (CVD), atherosclerosis is featured as a chronic inflammation. Hypercholesterolemia is an independent risk factor for CVD. Accumulated studies have shown that hypercholesterolemia is associated with myeloid cell expansion, which stimulates innate and adaptive immune responses, strengthens inflammation, and accelerates atherosclerosis progression. Hematopoietic stem/progenitor cells (HSPC) in bone marrow (BM) expresses a panel of lipoprotein receptors to control cholesterol homeostasis. Deficiency of these receptors abrogates cellular cholesterol efflux, resulting in HSPC proliferation and differentiation in hypercholesterolemic mice. Reduction of the cholesterol level in the lipid rafts by infusion of reconstituted high-density lipoprotein (HDL) or its major apolipoprotein, apoA-I, reverses hypercholesterolemia-induced HSPC expansion. Apart from impaired cholesterol metabolism, inhibition of reactive oxygen species production suppresses HSPC activation and leukocytosis. These data indicate that the mechanisms underlying the effects of hypercholesterolemia on HSPC proliferation and differentiation could be multifaceted. Furthermore, dyslipidemia also regulates HSPC-neighboring cells, resulting in HSPC mobilization. In the article, we review how hypercholesterolemia evokes HSPC activation and mobilization directly or via its modification of BM microenvironment. We hope this review will bring light to finding key molecules to control HSPC expansion, inflammation, and atherosclerosis for the treatment of CVD.

## 1. Preface

Cardiovascular disease (CVD) has remained the number one cause of mortality in the world for decades. The pathological basis of CVD is atherosclerosis, which is featured as atherosclerotic plaques in the artery wall leading to the restriction of blood flow. From the pathophysiological aspect, atherosclerosis initiates from disrupted endothelium which allows circulating apolipoprotein B (apoB)-containing lipoproteins to penetrate and accumulate in subendothelium where they further undergo chemical modification. Modified lipoproteins, particularly, oxidized low-density lipoprotein (LDLs), promote the proinflammatory phenotype of endothelial cells for increased vascular cell adhesion protein 1 (VCAM1) and intercellular adhesion molecule 1 (ICAM1) expression and proinflammatory cytokine production, all of which attract circulating white blood cells homing to the lesion site [1,2,3,4]. Following infiltration into the lesion site, monocytes, dendritic cells and T lymphocytes uptake fat and cholesterol to become foam cells that aggravate the inflammation cascade [5,6,7]. In the meantime, medical smooth muscle are activated for proliferation and migration to participate plaque formation and progression [8]. As a result, atherosclerotic plaque is formed with cellular components, such as white blood cells and smooth muscle cells, as well as acellular components, including cholesterol and collagen, leading to the restriction of blood flow.

Compelling evidence has demonstrated how risk factors, such as hypercholesterolemia, provoke inflammation and reinforce the initiation and progression of atherosclerosis. Interestingly, recent studies reported that hypercholesterolemia has an impact on hematopoietic stem/progenitor cells (HSPC) in the bone marrow (BM) niche to strengthen inflammation. In this article, we will explore how hypercholesterolemia orchestrates inflammatory cells, in particular, HSPC in BM in the pathogenesis of atherosclerosis.

## 2. Hypercholesterolemia Facilitates Inflammation

Physically, endothelial cells in arteries are exposed to unsteady flow because the velocity of blood waves during cardiac cycle. Endothelial cells sense the mechanic changes of blood flow and respond for adaptation, in which Krüppel-like factor 2 (KLF2) and endothelial nitric oxide synthase (eNOS) are fluid shear stress-responsive proteins and play crucial roles in the preservation of endothelium function [9]. However, in the regions close to bifurcation and curve of arteries, the endothelial phenotype is altered in response to the disturbed flow. They display a proinflammatory profile as featured by the activation of nuclear factor kappa B (NF-κB) [10,11], production of proinflammatory cytokines [12], and adhesion molecules [13,14], with reduced eNOS expression [10,11,13,14]. To be addressed, the sites with disturbed flow, also called “atherosusceptible sites” do not progress to significant inflammation and atherosclerosis until risk factors add onto it. In the text below, we focus on how hypercholesterolemia speeds up the development of atherosclerosis.

Indeed, myeloid cells, endothelial cells, and lymphocytes in atherosclerotic plaques are all affected by hypercholesterolemia. Numerous in vitro and in vivo mice studies have illustrated that excessive accumulation of intracellular cholesterol [15,16] and oxidized LDL [17] promote endothelial cell apoptosis and dysfunction. Although the molecular pathways might differ, elevated reactive oxygen species (ROS) production, with reduced nitric oxide level and bioavailability, are consistently involved in hypercholesterolemia-induced endothelial cell apoptosis [15,18,19]. Following endothelial cell apoptosis and dysfunction, a series of proinflammatory cytokines including tumor necrosis factor-α (TNF-α), monocyte chemoattractant protein-1 (MCP-1), C-reactive protein and adhesion molecules such as VCAM1 and ICAM1 are induced, which trigger white blood cell recruitment toward activated endothelial cells [20,21,22,23,24].

Using the high-speed multichannel epifluorescence and 2-photon laser scanning microscopy (TPLSM), inflammatory cells that recruit to lesion site could be visualized in living apoE^−/−^ mice after injection of fluorescently-labeled antibodies against monocytes, neutrophils, T lymphocytes or activated platelets, separately. This study elaborately demonstrated the timing and dynamics of different leukocyte subsets rolling to carotid bifurcation: (1) myeloid cells started to adhere to the carotid bifurcation after 10 days of high-fat diet (HFD); (2) the number of rolling neutrophils increased over time after two and six weeks of HFD, whereas monocytes number remained similar as both time points, but decreased after six weeks of HFD; and (3) T lymphocytes started rolling after six weeks of HFD. In addition, they found that activated platelets were adhered to the plaque via interaction of previously-tethered monocytes and neutrophils [25].

Following recruitment, neutrophils, monocytes, T lymphocytes, and platelets synergistically elicit inflammation and contribute substantially to atherosclerosis progression. Macrophages uptake lipoproteins and become foam cells in the plaque. As the hallmark of atherosclerotic plaque, foam cells are involved in all stages of atherosclerosis, from the initiation to progression of the plaque, to foam cell necrosis for plaque vulnerability and clinical features of atherosclerosis [26].

The activity of macrophages are regulated by neutrophils, T lymphocytes, and platelets. Physically, neutrophils are the first cells to home in on the infection site where they secrete granule proteins to clear microorganisms [27]. Depletion of neutrophils in apoE^−/−^ mice on HFD results in reduced plaque size with decrease of monocyte/macrophage and dendritic cell number in the aortic lysate, implying the role of neutrophils in the guidance of monocyte and dendritic cell rolling to the lesion in the development of atherosclerosis [28]. Th1 cells could regulate macrophage activity by releasing inflammatory cytokines and activating of mast cells for IgE production [29,30]. The pathological roles of activated platelets in atherosclerosis are multidimensional. Platelets interact with leukocytes via receptor and ligand interaction [31,32,33]. Once platelets adhere to inflamed endothelial cells, these interactions facilitate leukocytes extravasation and infiltration into plaque. Moreover, platelets could regulate leukocyte activity for phagocytosis [34,35], generation of reactive oxygen species [36] and monocyte differentiation to macrophages [37].

Hypercholesterolemia also interplays with immunity in atherosclerosis. Toll-like receptors (TLRs) sense danger signals from pathogens and activate innate immune response for defense. Hypercholesterolemia activates TLR4 pathways, which suppresses the activity of Liver X Receptor (LXR) on it target genes, leading to impaired cholesterol efflux in macrophages [38,39]. Oxidative lipoproteins and ROS are both components to provoke innate immunity. Dendritic cells process and present epitopes/major histocompatibility complex (MHC) complexes of oxidized lipoproteins and heat shock protein 70 to naïve T cells, which stimulate effector T cell expansion and differentiation to participate inflammation [40,41].

For decades, there is a notion that leukocyte count predicts the incidence of cardiovascular events [42,43]. When looking for insight into differential white blood cell counts, circulating monocytes and neutrophils are positively associated with the occurrence and progression of CVD [44,45]. In line with these reports, hypercholesterolemia is tightly associated with monocytosis and neutrophila [46], whereas Statins inhibits hypercholesterolemia-induced leukocytosis and reduces the amount of atherogenic inflammatory cells [46]. Recently, other groups and we have elucidated that hypercholesterolemia promotes hematopoietic stem/progenitor cell (HSPC) proliferation and differentiation, resulting in leukocytosis and plaque progression in hypercholesterolemic apoE^−/−^ [47,48], LDLr^−/−^ [48,49], and SR-BI^−/−^ [50] mice. Before we review how hypercholesterolemia modulates HSPC, we first briefly review the biology of HSPC.

## 3. Hematopoietic Stem/Progenitor Cells (HSPC) Biology

Despite controversy, hematopoiesis is comprised of two stages: “primitive hematopoiesis” in the yolk sac and “definitive hematopoiesis” in the aorta-gonad-mesonephros (AGM). Later on, hematopoiesis moves from AGM to the placenta, fetal liver at E11 and, finally, migrates to BM around birth [51,52,53]. Fetal liver and BM are both major sites of hematopoiesis, however, hematopoietic stem cells (HSC) in fetal liver are much more proliferative than HSC in BM, suggesting different metabolic demands. By performing RNA-Seq analysis of E14.5 fetal liver and BM HSC, Manesia et al. showed that HSC in fetal liver contained more mitochondria, which resulted in more ROS production [54]. Paradoxically, when residing in the hypoxic BM niche, 90% HSCs remains quiescent with limited ROS production [54]. The quiescent HSCs are located in the endosteal niche, whereas dividing HSCs could be found in the perivascular niche. The hypoxic microenvironment is favorable for maintaining the self-renewal capacity and function of HSPC [55,56].

Despite HSC being a small population, accounting for around 0.001% in total BM cells, they fulfill all of the characteristics of stem cells: the capacity of self-renewal in the generation of two HSCs and the ability of giving rise to all types of blood cells throughout life. The transition from quiescence to activation stages, and the mobilization of HSPC from BM to blood, are highly organized by intrinsic factors, extrinsic factors, receptors in HSPC, and surrounding cells in the niche. The stimuli for HSPC activation include chronic bacterial infection [57,58,59], psychosocial stress [60], injury, such as myocardial infarction [61], and hypercholesterolemia [47,48,50,62].

Except the intrinsic and extrinsic factors, HSPC activation and retention are modulated by the BM niche where HSPC are located. The supportive roles of BM niche on HSPC biology could be summarized below: (1) BM stromal cells and macrophages could produce hematopoietic cytokines such as thrombopoietin (TPO) [63], stem cell factor (c-Kit ligand) [64], and granulocyte colony-stimulating factor [65,66] to support hematopoiesis; (2) endothelial cells preserve vasculature and interact with HSPC via the Tie2/angiopoietin-1 axis, angiopoietin-like proteins, and vascular endothelial growth factor (VEGF) signaling to assist HSC regeneration [67,68,69,70]; (3) retention of HSPC in the niche is mediated by its interaction with endothelial cells and stromal cells via integrins and C–X–C chemokine receptor type 4 (CXCR4)/stromal-derived-factor-1 (SDF-1) [71,72,73]. Thus, it is not surprising that disturbing the stability of BM niche could substantially change the fate of HSPC.

Among all the regulatory factors, ROS is the essential one for HSPC activation. It is not just a byproduct of oxidative stress, ROS accompanies HSPC to enter cell cycle for proliferation and differentiation [74,75,76]. Although it is hard to discriminate whether increased ROS production in HSPC is the cause or consequence of HSPC proliferation, inhibition of ROS production rescues HSPC exhaustion and senescence and regains the self-renewal capacity of HSPC [77].

Except circulating between BM and blood, HSPC are also found in the lung, liver, and spleen. It is not known when they arrive there and the origins of these HSPC during hematopoiesis. Nevertheless, these resident HSPC have been proven to differentiate into lymphocytes for immune surveillance [78].

## 4. Hypercholesterolemia Induces HSPC Proliferation and Differentiation

Like mature cells, intracellular cholesterol homeostasis in HSPC mainly replies on ATP-binding cassette transporter (ABCA1), the ATP-binding cassette sub-family G member 1(ABCG1), scavenger receptor type B-I (SR-BI), and apolipoprotein E (apoE) on the surface of these cells. In the absence of the lipoprotein receptors, diet-induced hypercholesterolemia leads to HSPC proliferation and differentiation in the BM niche, contributing substantially to leukocytosis in the peripheral blood [47,50,62]. When exploring mechanisms, Yves-Charvet et al. showed that deficiency of ABCA1 and ABCG1 promoted cholesterol accumulation in HSPC membrane, especially in the lipid rafts, which increased the expression of β subunit of IL-3/granulocyte-macrophage colony-stimulating factor (GM-CSF) receptors and enhanced the proliferative responses to IL-3 and GM-CSF [62]. Likewise, Murphy et al. reported that impaired cholesterol efflux in HSPC failed to activate E3-ubiquitin ligase, resulting in increased IL-3Rβ and M-CSF receptor expression in HSPC [47]. Ultimately, elevation of the IL3/IL3R axis, M-CSF, and GM-CSF signaling skewed HSPC differentiation for myeloid lineage production. Consistent with these findings, Gao, et al. observed the expansion of HSPC, common myeloid progenitors and granulocyte-macrophage progenitors in BM of SR-BI^−/−^ mice on HFD [50]. Nonetheless, infusion of purified apoA-I or reconstituted HDL consistently reduced HSPC proliferation in these models, suggesting the importance of cholesterol homeostasis in controlling HSPC biology [47,50,62].

As described above, platelets play a key role in atherosclerosis. The biology of megakaryocyte progenitors (MKP) is tightly controlled by the turnover of TPO receptors, i.e., myeloproliferative leukemia protein (c-MPL) on MKP. In hypercholesterolemia, MKP in BM are challenged with increased cholesterol loading and impaired cholesterol efflux may develop in the absence of HDL receptor(s). Increased cellular cholesterol content increases c-MPL expression but decreases c-CBL (named after Casitas B-lineage Lymphoma) phosphorylation. The decreased c-CBL phosphorylation jeopardizes the negative feedback regulation of c-MPL by reduced ubiquitination and proteasomal degradation of c-MPL. Eventually, MKP are activated to proliferate in order to accelerate platelet production [79].

## 5. Reactive Oxygen Species (ROS) Reduction Suppresses HSPC Expansion

Is the impaired cholesterol efflux the solo mechanism for hypercholesterolemia-induced HSPC activation? The answer seems to be negative. When SR-BI^−/−^ and LDLr^−/−^ apoA^−/−^ mice fed on HFD received ROS inhibitor *N*-acetyl-l-cysteine (NAC), NAC reversed hypercholesterolemia-induced HSPC expansion, leukocytosis, and atherosclerosis progression in SR-BI^−/−^ and LDLr^−/−^ apoA-I^−/−^ mice. Nevertheless, NAC treatment did not alter serum cholesterol level in the mice [50]. In parallel, hypercholesterolemia, including LDL stimulates endothelial cells and myeloid cells for ROS production [80], posing HSPC in an oxidative microenvironment to accelerate their expansion. These data collectively indicate that ROS could be an independent regulator for HSPC proliferation and differentiation.

## 6. Hypercholesterolemia Modifies Bone Marrow (BM) Microenvironment for HSPC Mobilization

In addition to HSPC proliferation in the BM niche, the frequency of circulating HSPC is also increased in hypercholesterolemia mice, indicating enhanced HSPC mobilization. HSPC are in communication with different cell types in BM microenvironment via integrins and the CXCR4/SDF axis, all of which maintain HSPC in the cavity of BM. How does hypercholesterolemia influence the BM microenvironment for HSPC mobilization? Histological analysis reveals that megakaryocytes form large clusters in close to BM sinusoidal vessels [81], implicating a modified situation between thrombolysis and hematopoietic equilibrium. Gomez et al. further showed that hypercholesterolemia distorted SDF gradient between blood and BM, leading to HSPC mobilization [81]. Consistent with this study, Westerterp et al. further delicately delineated the underlying mechanisms how hypercholesterolemia promoted HSPC mobilization in ABCA1^−/−^ ABCG1^−/−^ mice [82]. They found that impaired cholesterol efflux increased extramedullary hematopoiesis with increased production of IL-23/IL-17/G-CSF in splenic macrophages and dendritic cells. They further demonstrated that hypercholesterolemia indirectly modified the structure of BM microenvironments by reduction of osteoblast numbers, especially, *N*-Cadhern^+^ osteoblasts and Nestin^+^ mesenchymal stem cells. As the main source of SDF, the decreased number of osteoblasts and Nestin^+^ mesenchymal stem cells resulted in reduced SDF production. Taken together, increased IL23/IL17/G-CSF production in the peripheral blood and reduced SDF in BM synergistically foster HSPC mobilization.

## 7. Hypercholesterolemia Potentiates HSPC Homing to Lesion Site

So far, a strong link has been verified between hypercholesterolemia, HSPC expansion, inflammation and atherosclerosis. Although HSPC reside in the BM niche, a minority of HSPC are trafficking among BM and blood and could home to tissues where they become resident cells for immune defense [78,83]. Therefore, questions arose: could BM-derived HSPC directly participate atherosclerosis? Whether and how does hypercholesterolemia impact on HSPC function?

To explore the questions, Wang et al. screened integrins’ expression on HSPC in LDLr^−/−^ mice fed on chow or HFD. By flow cytometry, increased integrin β_2_ expression was detected in HSPC of hypercholesterolemia LDLr^−/−^ mice compared with controls [84]. When fluorescently-labeled lineage-negative cells were injected to the recipients baring a complete ligation in the right carotid artery, Lineage-Sca-1^+^cKit^+^ HSPC were identified homing to the injury site by flow cytometry. The nature of homed HSPC was further confirmed by their potential in BM reconstitution upon injection into lethally irradiated recipients. Due to the technical limitation, it is not feasible to trace the fate of homed HSPC in the ligated artery. However, one assumption could be drawn that hypercholesterolemia could modify HSPC motility and enhance recruitment activity to contribute to atherosclerosis.

## 8. High-Density Lipoprotein and HSPC

Distinct from very low-density lipoprotein (VLDL) and LDL, high-density lipoproteins (HDLs) and its major apolipoprotein A-I (apoA-I) are inversely related to the risk of CVD [85]. The mechanisms underlying the beneficial effects of HDLs and apoA-I include reverse cholesterol transport, maintenance of endothelium integrity, and suppression of inflammation [86,87,88,89].

**Figure 1:**
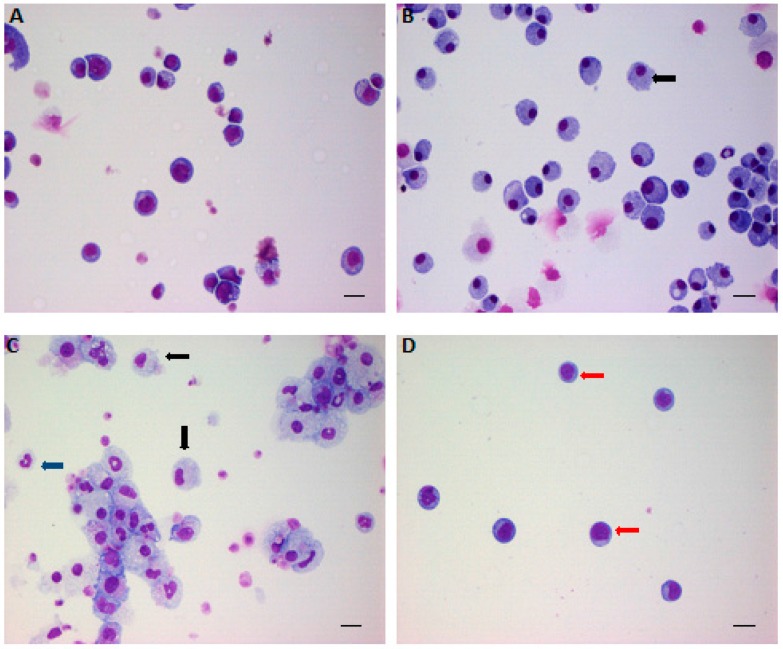
The effects of low-density lipoprotein (LDL) and high-density lipoprotein (HDL) on hematopoietic stem/progenitor cell (HSPC) expansion and differentiation. Lineage^-^ Sca-1^+^ cKit^+^ cells were sorted out by fluorescence-activated cell sorting (FACS) and cultured in serum-free medium in the presence of BSA (**A**); granulocyte-macrophage colony-stimulating factor (GM-CSF) (**B**); or LDL (**C**); and LDL plus HDL (**D**) for 14 days and then collected by cytospin for Giemsa staining. Red arrow indicates quiescent HSPC; black arrow indicates promonocytes; blue arrow indicates granulocytes. Scale bar: 20 μm. Adapted from Feng et al, PLoS ONE 2012.

HDL-mediated cholesterol efflux serves as fundamental machinery to achieve cholesterol homeostasis in cells. HSPC are not the exception. In vitro, addition of HDLs to cultured Lineage^-^Sca-1^+^cKit^+^ HSPC retains the quiescence status and prohibits LDL-induced HSPC proliferation and differentiation, therefore, limiting the production of atherogenic myeloid cells [48,90] (Figure 1). In vivo, infusion of reconstitute HDL or purified apoA-I abolishes hypercholesterolemia-induced HSPC expansion and differentiation or MKP proliferation and, thus, suppresses inflammation and atherosclerosis progression [47,50,79]. Improved cholesterol metabolism or reduced ROS content is detected in these models. The detailed mechanisms how HDLs and apoA-I regulate HSPC is largely unknown. Neither is clear how HDLs orchestrate HSPC-neighboring cells in the BM niche for HSPC maintenance.

## 9. Hyperglycemia, Obesity, and Myelopoiesis

Except hypercholesterolemia, skewed myeloid cell expansion is also observed in diabetic mice and obese subjects [91,92]. Studies of type 1 diabetic (T1D) mice uncovered that hyperglycemia stimulates neutrophils to produce S100A8/A9. Once released, S100A1/A9 acts on the receptor for advanced glycation endproducts (RAGE) on BM common myeloid progenitors (CMP) and macrophages to activate NF-κB transcription for M-CSF and GM-CSF production, both of which trigger CMP and granulocyte-macrophage progenitor (GMP) proliferation for myelopoiesis [93].

Adipose tissues are another source for S100A8/A9 production. In obese ob/ob mice, excessive visceral adipose tissues produce large amounts of S100A8/A9 that promotes Toll-like receptor 4 (TLR4)-Myeloid differentiation factor 88 (MyD88)signaling in adipose tissue macrophages for NACHT, LRR and PYD domains-containing protein 3 (NLRP3) inflammasome activation and IL-1β production. Through circulation, IL-1β reaches BM where it engages to IL-1R on CMP and GMP to drive myelopoiesis, leading to monocytosis and neutrophilia [94]. The intimate communication between adipose tissue macrophages and BM HSPC is also illustrated in wide-type mice fed on HFD in which MyD88 mediates HSPC proliferation for increased CMP and GMP, resulting in myelopoiesis. To be noted, different from hypercholesterolemia, the frequency of HSPC in BM of T1D mice or ob/ob mice is not altered [93,94]. It will be of great interest to explore why autonomous obesity and T1D affect CMP and GMP, but not HSPC.

## 10. Summary and Perspectives

**Figure 2:**
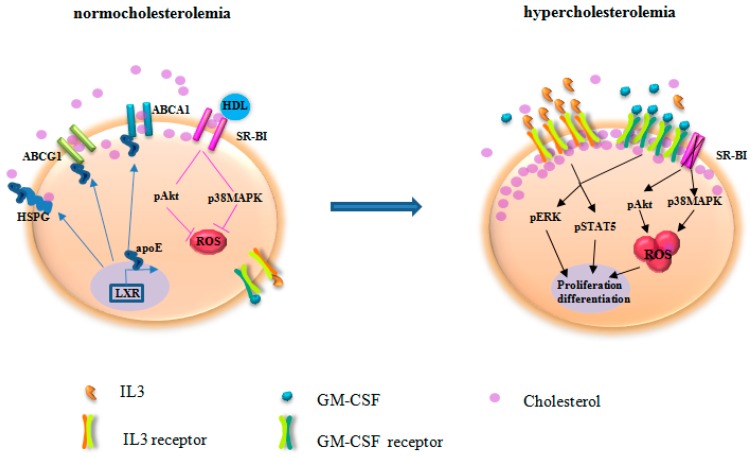
The mechanisms underlying the impact of hypercholesterolemia on HSPC. Under normocholesterolemia, ATP-binding cassette transporter (ABCA1), ATP-binding cassette sub-family G member 1(ABCG1), and scavenger receptor type B-I (SR-BI) facilitate cholesterol efflux. After secretion, apolipoprotein E (apoE) binds to heparin sulfate proteoglycans (HSPG) for cholesterol removal. Furthermore, apoE and apoE-containing HDL interact with ABCA1 and ABCG1 for reverse cholesterol transport. apoE, ABCA1, and ABCG1 expression are regulated by liver X receptor (LXR). HDL acts via SR-BI to control Akt and p38 mitogen-activated protein kinases (p38MAPK) phosphorylation and reactive oxygen species (ROS) production. As the net result, cholesterol homeostasis is maintained in HSPC. Deficiency of ABCA1, ABCG1, apoE, and SR-BI disrupts cholesterol efflux. Hypercholesterolemia accelerates impaired cholesterol efflux, leading to enriched cholesterol accumulation in the lipid raft. Thus, the expression of the common β subunit of the IL-3/GM-CSF receptors is increased, resulting in elevated response to IL-3 and GM-CSF for HSPC proliferation. In parallel, SR-BI deficiency abrogates the regulation of HDL on ROS production. Therefore, HSPC are activated for proliferation and differentiation. The skewed HSPC differentiation to myeloid cells produces IL-3 and GM-CSF that further potentiate HSPC activation. pERK, phosphorylated extracellular signal-regulated kinase; pSTAT5, phosphorylated signal transducer and activator of transcription 5.

The intimate relationship between inflammation and atherosclerosis progression has been confirmed. Suppression of inflammation as an adjunct factor for risk factor, is being tested in clinical trials and the results will come out next year [95,96]. As an independent risk factor of CVD, hypercholesterolemia stimulates HSPC proliferation and differentiation in BM, enhances ROS production in HSPC, increases the pool of inflammatory cells in peripheral blood and, thus, aggravates atherosclerosis. The mechanisms underlying the impact of hypercholesterolemia on HSPC are depicted in Figure 2. Improved cholesterol homeostasis and inhibition of ROS production serve as two pillars in the suppression of HSPC expansion and inflammation in the setting of hypercholesterolemia. Stabilization of BM niche may assist HSPC quiescence status and inhibition of inflammatory cell production. Thus, there is a need to search for key regulators that retain HSPC quiescence and function for inflammatory control when combating hypercholesterolemia-induced atherosclerosis.
